# Skeletal Muscle PGC‐1α Remodels Mitochondrial Phospholipidome but Does Not Alter Energy Efficiency for ATP Synthesis

**DOI:** 10.1002/jcsm.70090

**Published:** 2025-10-09

**Authors:** Takuya Karasawa, Ran Hee Choi, Cesar A. Meza, Shinya Watanabe, J. Alan Maschek, Linda S. Nikolova, James E. Cox, Katsuhiko Funai

**Affiliations:** ^1^ Diabetes & Metabolism Research Center University of Utah Salt Lake City Utah USA; ^2^ Department of Nutrition & Integrative Physiology University of Utah Salt Lake City Utah USA; ^3^ Research Institute for Sport Science Nippon Sport Science University Tokyo Japan; ^4^ Metabolomics Core Research Facility University of Utah Salt Lake City Utah USA; ^5^ Electron Microscopy Core Facility University of Utah Salt Lake City Utah USA

**Keywords:** exercise, mitochondria, phospholipids, skeletal muscle

## Abstract

**Background:**

The coupling of oxygen consumption to ATP synthesis via oxidative phosphorylation (OXPHOS) is central for cellular energy homeostasis. Some studies suggest exercise training increases the efficiency of ATP synthesis, but the molecular mechanisms are unclear. We have previously shown that exercise remodels the lipid composition of mitochondrial membranes, and some of these changes in mitochondrial lipids might influence OXPHOS efficiency (ATP produced per O_2_ consumed, referred to as P/O). Peroxisome proliferator‐activated receptor gamma coactivator‐1 alpha (PGC‐1α) is a transcriptional co‐activator that coordinately regulates exercise‐induced adaptations, including mitochondria. We hypothesized that increased PGC‐1α activity might remodel mitochondrial membrane lipids and promote energy efficiency.

**Methods:**

Mice with skeletal muscle‐specific overexpression of PGC‐1α (MCK‐PGC‐1α) and their wildtype littermates were used for this study. Lipid mass spectrometry and quantitative PCR were used to assess muscle mitochondrial lipid composition and their biosynthesis pathway. The abundance of OXPHOS enzymes was determined by Western blotting. High‐resolution respirometry and fluorometry analyses were performed to characterize mitochondrial bioenergetics (ATP production, O_2_ consumption and P/O) for permeabilized fibres and isolated mitochondria. Respiratory supercomplexes were assessed by blue native PAGE.

**Results:**

Lipidomic analyses of skeletal muscle mitochondria from wildtype and MCK‐PGC‐1α mice revealed that PGC‐1α increases the concentrations of cone‐shaped lipids such as phosphatidylethanolamine (PE; +25%, *p* < 0.0001), cardiolipin (CL; +184%, *p* < 0.0001) and lysophospholipids (+34%–94%, all *p* < 0.01), while decreasing the concentrations of phosphatidylcholine (PC; −4%, *p* = 0.0020), phosphatidylinositol (PI; −17%, *p* < 0.0001) and phosphatidic acid (PA; −35%, *p* < 0.0001). However, while PGC‐1α overexpression increased the abundance of OXPHOS enzymes (two‐ to fourfold, *p* < 0.0001), the rate of O_2_ consumption (1.5‐fold, *p* = 0.0030), or the respiratory supercomplexes (~1.5‐fold, *p* < 0.01), P/O values were unaffected by PGC‐1α overexpression in permeabilized fibres or isolated mitochondria.

**Conclusions:**

Collectively, overexpression of PGC‐1α promotes the biosynthesis of mitochondrial PE and CL, but neither PGC‐1α nor the mitochondrial membrane lipid remodelling induced in MCK‐PGC‐1α mice is sufficient to increase the efficiency of mitochondrial ATP synthesis. These findings indicate that PGC‐1α‐dependent mechanisms or changes in mitochondrial membrane lipids may be insufficient to alter P/O. While muscles from MCK‐PGC‐1α mice are known not to completely phenocopy adaptations with exercise training, our findings also highlight that there is a need to examine whether exercise training indeed improves P/O in mouse skeletal muscle.

## 1 Introduction

Skeletal muscle is a large contributor to resting whole‐body energy expenditure, with its energy demand increasing significantly during physical activity [1]. The coupling of oxygen consumption to ATP synthesis through oxidative phosphorylation (OXPHOS) is a critical step in converting energy substrates into ATP for cellular activity. Previous studies have suggested that mitochondrial energy efficiency for ATP synthesis may increase in response to a sustained high energy demand, such as during exercise [2, 3]. In contrast, reduced mitochondrial energy efficiency in skeletal muscle is observed in aging as well as with chronic kidney disease [4, 5]. While mechanisms that control mitochondrial volume and respiratory function in skeletal muscle are almost exhaustively studied [6, 7], there is very little literature on the regulation of mitochondrial OXPHOS energy efficiency [2, 8].

Peroxisome proliferator‐activated receptor‐γ coactivator‐1α (PGC‐1α) is a transcription coactivator that plays a key role in the regulation of mitochondrial adaptations in skeletal muscle [9, 10, 11]. The expression of PGC‐1α is known to increase in response to metabolic stress, such as exercise, and decline with aging [10, 11, 12]. Overexpression of PGC‐1α in skeletal muscle robustly increases mitochondrial volume, resulting in enhanced capacity for respiration and ATP synthesis [13, 14, 15, 16, 17]. Conversely, depletion of PGC‐1α results in reduced mitochondrial volume and enzymes of OXPHOS, leading to decreased respiration and exercise capacity [18, 19, 20, 21]. Loss‐of‐function experiments further indicate that PGC‐1α is essential for mitochondrial cristae architecture, likely affecting bioenergetics [22]. Although no studies have examined the effect of PGC‐1α on the energy efficiency of skeletal muscle OXPHOS, it is reasonable to speculate that PGC‐1α may alter skeletal muscle mitochondrial energy efficiency.

**FIGURE 1:**
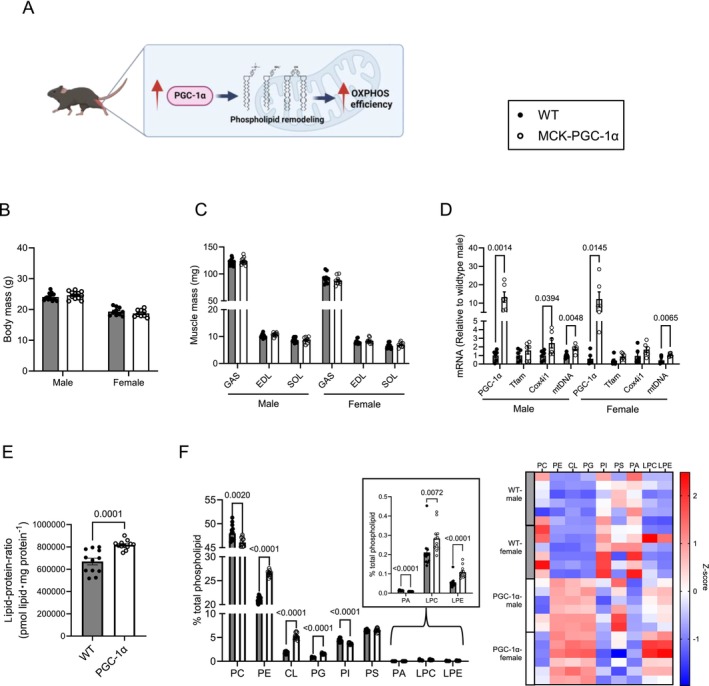
Overexpression of PGC‐1α alters muscle mitochondrial phospholipid composition (A) Schematic illustration of our hypothesis that PGC‐1α increases skeletal muscle OXPHOS efficiency. (B) Body mass of male and female WT and MCK‐PGC‐1α mice (WT *n* = 24 [male:female = 14:10], MCK‐PGC‐1α *n* = 21 [male:female = 12:9]). (C) Gastrocnemius (GAS), extensor digitorum longus (EDL), soleus (SOL) muscle mass (WT *n* = 24 [male:female = 14:10], MCK‐PGC‐1α *n* = 21 [male:female = 12:9]). (D) mRNA levels of Pgc‐1α and mitochondrial biogenesis markers in gastrocnemius muscle (WT *n* = 12 [male:female = 6:6], MCK‐PGC‐1α *n* = 12 [male:female = 6:6]). (E) Lipid‐protein‐ratio, derived by dividing the sum of all mitochondrial phospholipids by mitochondrial protein. A surrogate for how concentrated proteins is in the mitochondrial membranes (WT *n* = 12 [male:female = 6:6], MCK‐PGC‐1α *n* = 12 [male:female = 6:6]). (F) Relative abundance of each mitochondrial lipid class and its corresponding heatmap (WT *n* = 12 [male:female = 6:6], MCK‐PGC‐1α *n* = 12 [male:female = 6:6]). Data are represented as mean ± SEM.

Mitochondria are composed of bilayer membranes that primarily consist of phospholipids. In particular, the lipid composition of the inner mitochondrial membrane (IMM) directly influences OXPHOS by modulating protein function via lipid‐protein interactions, membrane properties and cristae morphology [23]. Cone‐shaped phospholipids such as phosphatidylethanolamine (PE) and cardiolipin (CL) are concentrated in the IMM presumably to induce curvatures that are found in the cristae where OXPHOS enzymes reside [23, 24]. We previously reported that mitochondrial PE and CL are elevated with exercise and influence OXPHOS activity and likely efficiency [25]. Our recent study also suggests that weight loss‐induced improvement in OXPHOS energy efficiency is associated with an increase in the concentration of tetralinoleoyl‐CL in mitochondria [8]. In contrast, CL synthase expression and CL abundance are downregulated in aged skeletal muscle [26]. Overexpression of PGC‐1α increases several species of PE in skeletal muscle [27], while depletion of PGC‐1α decreases total CL abundance in the heart [28]. Nonetheless, these changes represent an alteration in the total cellular lipidome rather than changes that are specific to mitochondria. The purpose of the current study was to test the hypothesis that PGC‐1α remodels the skeletal muscle mitochondrial membrane lipid composition, and that these changes would coincide with improved efficiency of mitochondrial ATP synthesis (Figure 1A).

## 2 Materials and Methods

### 2.1 Animals

Ten‐ to twelve‐week‐old wildtype and MCK‐PGC‐1α (RRID:IMSR_JAX:008231; PGC‐1α overexpression under the control of the muscle creatine kinase promoter, kindly provided by Dr. Bruce Spiegelman) littermates of both sexes were used for this study [10]. Mice were housed under a 12‐h light/dark cycle in a temperature‐controlled room. For terminal experiments, mice were given an intraperitoneal injection of 80 mg/kg ketamine and 10 mg/kg xylazine, after which extensor digitorum longus (EDL), soleus (SOL), gastrocnemius (GAS), and tibialis anterior (TA) muscles were harvested. All experimental procedures were approved by the University of Utah Institutional Animal Care and Use Committee.

### 2.2 Isolation of Skeletal Muscle Mitochondria

Muscle mitochondrial isolation was performed as previously described [25]. Briefly, GAS muscle from freshly sacrificed mice was finely minced in an ice‐cold mitochondrial isolation medium (MIM: 0.3 M sucrose, 10 mM 4‐(2‐hydroxyethyl)‐1‐piperazineethanesulfonic acid [HEPES], 1 mM ethylene glycol tetraacetic acid [EGTA], pH 7.1) with bovine serum albumin (BSA: 1 mg/mL). The mixture was homogenized using a TH‐01 homogenizer (Omni International). Homogenates were centrifuged at 800*g* for 10 min at 4°C twice. Each time, the supernatant was collected and transferred to a new tube. The supernatant was centrifuged at 12 000*g* for 10 min at 4°C. The supernatant was discarded, and the pellet was resuspended in 1 mL MIM. The resuspension was centrifuged once more at 12 000*g* for 10 min at 4°C, and the pellet was resuspended in 100 μL MIM.

### 2.3 Mitochondrial Lipid Mass Spectrometry

Mitochondrial phospholipids were extracted from isolated mitochondria using a modified Matyash lipid extraction protocol (Data S2: [S1]). A mixture of ice‐cold methyl‐tert‐butyl ether (MTBE), methanol and internal standards (EQUISPLASH Mix [Avanti Polar Lipids 330731] and Cardiolipin Mix I [Avanti Polar Lipids LM6003]) was added to 50 μg of protein from isolated mitochondria from GAS muscle. Samples were vortexed and sonicated for 1 min before being incubated on ice for 15 min. During this time, samples were vortexed every 5 min. H_2_O was added, and the samples were again incubated on ice for 15 min with vortexing every 5 min. The samples were centrifuged at 12 000*g* for 10 min. The organic (upper) layer was collected, and the aqueous layer was re‐extracted with 1 mL of 10:3:2.5 (v/v/v) MTBE/methanol/H_2_O. The MTBE layers were combined for untargeted lipidomic analysis and dried under vacuum. Lipid extracts were reconstituted in 300 μL of 8:2:2 (v/v/v) IPA/ACN/H_2_O. Lipidomics analysis was conducted at the Metabolomics Core at the University of Utah using liquid chromatography–mass spectrometry on an Agilent 6545 ultraperformance liquid chromatography‐quadrupole time‐of‐flight mass spectrometer.

### 2.4 Preparation for Permeabilized Muscle Fibre Bundles (PmFBs)

PmFBs were prepared as previously described [8]. Briefly, a small portion of freshly dissected red portion of the GAS muscle was placed in buffer X (7.23 mM K_2_EGTA, 2.77 mM Ca K_2_EGTA, 20 mM imidazole, 20 mM taurine, 5.7 mM ATP, 14.3 mM phosphocreatine, 6.56 mM MgCl_2_·6H_2_O and 50 mM 2‐(N‐Morpholino) ethanesulfonic acid potassium salt [K‐MES] [pH 7.4]). Fibre bundles were separated and permeabilized for 30 min at 4°C with 30 μg/mL saponin. After permeabilization, fibre bundles were incubated in buffer Z (105 mM K‐MES, 30 mM KCl, 10 mM K_2_HPO_4_, 5 mM MgCl_2_·6H_2_O, 0.5 mg/mL BSA and 1 mM EGTA [pH 7.4]) with 0.5 mM pyruvate and 0.2 mM malate for 15 min and briefly washed with buffer Z. PmFB samples were placed in buffer Z until analysis.

### 2.5 High‐Resolution Respirometry and Fluorometry

O_2_ consumption of both the PmFB and isolated muscle mitochondria from freshly dissected GAS muscle was measured using Oroboros Oxygraph‐2k (Oroboros Instruments). ATP production was assessed using Fluorolog‐QM (Horiba Scientific) by enzymatically coupling ATP production to NADPH synthesis, as previously described (Data S2: [S2]). Both experiments were conducted in buffer Z at 37°C. O_2_ consumption and ATP production were stimulated by treating PmFB or isolated muscle mitochondria with 0.5 mM malate, 5 mM pyruvate, 5 mM glutamate, 10 mM succinate and successive additions of ADP (20, 200 and 2000 μM for PmFB; 2, 20 and 200 μM for isolated muscle mitochondria). In the PmFB experiments, 20 mM creatine monohydrate and 10 μM blebbistatin were added to buffer Z to inhibit myosin adenosine triphosphatases. The P/O ratio was determined by dividing ATP production by O_2_ consumption.

### 2.6 Quantitative PCR

The frozen GAS muscle was homogenized using TissueLyser II (Qiagen) in 1 mL of TRIzol (Thermo Fisher Scientific). Following homogenization, 200 μL of chloroform was added to the tube and mixed by inverting it 10 times. After 2 min, samples were centrifuged at 12 000*g* for 15 min at 4°C. The aqueous supernatant was collected and placed in a new tube containing 500 μL of 100% isopropyl alcohol. The mixture was inverted a few times and incubated for 10 min. The samples were centrifuged at 12 000*g* for 10 min at 4°C. The supernatant was carefully aspirated, and the pellet was washed with 75% ethyl alcohol and centrifuged at 7500*g* for 5 min. The supernatant was aspirated, and the pellet was air‐dried before being resuspended in diethylpyrocarbonate‐treated water. RNA was reverse‐transcribed using an iScript cDNA synthesis kit (Bio‐Rad). For quantitative PCR, an equal amount of cDNA was placed in a 384‐well plate with SYBR Green (Thermo Fisher Scientific) and gene‐specific primers. The primer sequences used are shown in Table S1. Gene expression was analysed using a QuantStudio 12K Flex (Life Technologies) and was normalized to ribosomal protein L32.

### 2.7 Western Blotting

For whole muscle tissue analysis, the frozen GAS, EDL, SOL or TA muscle was homogenized using TH‐01 homogenizer in ice‐cold RIPA buffer (Thermo Fisher Scientific) supplemented with protease inhibitor cocktail (Thermo Fisher Scientific). Following homogenization, the samples were centrifuged at 12 000*g* for 10 min at 4°C. The protein concentration in the supernatant was then determined using the BCA Protein Assay Kit (Thermo Fisher Scientific). For protein analysis in isolated mitochondria, the previously prepared isolated muscle mitochondria, used for high‐resolution respirometry and fluorometry analysis, were utilized. Subsequent steps were identical for both sample types. Equal amounts of protein were mixed with Laemmli sample buffer (Bio‐Rad) and loaded onto a 4%–20% gradient SDS‐polyacrylamide gel (Bio‐Rad) for electrophoresis. The proteins were then transferred from the gel to polyvinylidene fluoride membranes (Thermo Fisher Scientific). The membranes were blocked with 3% skim milk in Tris‐buffered saline containing 0.1% Tween 20 (TBST) for 1 h at room temperature, followed by overnight incubation at 4°C with primary antibodies targeting glyceraldehyde‐3‐phosphate dehydrogenase (GAPDH) (Cell Signaling Technology, 2118, 1:1000), cytochrome c oxidase (COX) IV (Cell Signaling Technology, 4844, 1:1000), total OXPHOS (Abcam, MS604‐300, 1:1000) or citrate synthase (Abcam, Ab96600, 1:1000), mitofusin (MFN) 1 (proteintech, 13798‐1‐AP, 1:2000), MFN2 (proteintech, 12186‐1‐AP, 1:1000), optic atrophy 1 (OPA1) (Cell Signaling Technology, 80471, 1:1000), dynamin‐related protein 1 (DRP1) (proteintech, 12957‐1‐AP, 1:2000), sarcoplasmic/endoplasmic reticulum calcium ATPase (SERCA) 1 (Cell Signaling Technology, 12293, 1:1000), SERCA2 (Abcam, ab3625, 1:1000), myosin heavy chain (MHC) type IIa (Developmental Studies Hybridoma Bank [DSHB], SC‐71, 1:1000), MHC type IIb (DSHB, BF‐F3, 1:1000) and MHC type IIx (DSHB, 6H1, 1:1000). After washing three times with TBST, the membranes were incubated with secondary antibodies diluted 1:5000 in 1% skim milk for 1 h at room temperature. Following three washes in TBST and one in TBS, membranes were incubated with Western Lightning Plus‐ECL (PerkinElmer) and imaged using a ChemiDoc Imaging System (Bio‐Rad) and quantified with Image Lab software (Bio‐Rad). The membranes were stained with Ponceau S (Sigma‐Aldrich) to verify equal protein loading across lanes.

### 2.8 Blue Native PAGE

Mitochondrial supercomplex analysis was performed as previously described with modification (Data S2: [S3, S4]). Isolated mitochondria (25 μg) suspended in MIM were pelleted at 12 000*g* for 10 min and subsequently solubilized in 20 μL sample buffer (5 μL of 4x Native PAGE Sample Buffer, 2 μL 10% digitonin, 13 μL ddH_2_O per sample) for 20 min on ice and then centrifuged at 18 000*g* for 10 min at 4°C. Fifteen microlitres of the supernatant was collected and placed into a new tube and mixed with 1 μL of G‐250 sample additive (Thermo Fisher Scientific). The samples and protein ladder were loaded onto a Native PAGE 3%–12% Bis‐Tris Gel (Thermo Fisher Scientific). Dark blue cathode buffer (0.02% Coomassie Brilliant Blue dissolved in Native PAGE running buffer) was carefully added to the front of the gel box (Thermo Fisher Scientific) and Native PAGE running buffer (anode buffer) was carefully added to the back of the gel box, making sure not to mix. Electrophoresis was performed at 150 V for 45 min at 4°C. The dark blue cathode buffer was then replaced with light blue cathode buffer (dark blue cathode buffer and anode buffer 1:9) and run at 15 mA for 90 min at 4°C. Gels were subsequently stained with Coomassie Brilliant Blue staining solution (0.05% Coomassie Brilliant Blue, 50% methanol, 10% acetic acid) and washed in destaining solution (50% methanol, 7% acetic acid) followed by water to visualize the bound protein and ladder. The detection of protein was performed using a ChemiDoc Imaging System (Bio‐Rad) and quantified with Image Lab software (Bio‐Rad).

### 2.9 Electron Microscopy

Sample preparation for electron microscopy was performed as previously described [25]. Briefly, freshly dissected red part of the GAS muscles was placed in a fixative solution (1% glutaraldehyde, 2.5% paraformaldehyde, 100 mM cacodylate buffer pH 7.4, 6 mM CaCl_2_, 4.8% sucrose), sectioned into ~2‐mm pieces and stored at 4°C for 48 h. The Electron Microscopy Core at the University of Utah prepared the samples. The tissues were washed three times for 10 min each in 100 mM cacodylate buffer (pH 7.4) prior to secondary fixation with 2% osmium tetroxide for 1 h at room temperature. Osmium tetroxide was used as a secondary fixative, as it primarily reacts with lipid moieties and adds density and contrast to tissue, which cannot be achieved by aldehydes alone (Data S2: [S5]). The primary fixation first crosslinks proteins and carbohydrate molecules, and then osmium tetroxide crosslinks the lipids and helps stabilize various cell components. After secondary fixation, the samples were rinsed in cacodylate buffer for 5 min, followed by distilled water and then prestained with saturated uranyl acetate for 1 h at room temperature. Following prestaining, the samples were dehydrated in a graded ethanol series (2 × 15 min each: 30%, 50%, 70%, 95%; then 3 × 20 min each: 100%) and acetone (3 × 15‐min washes). The samples were infiltrated with EPON epoxy resin (5 h 30%, overnight 70%, 3 × 2‐h 40 min 100%, 100% fresh for embed). Polymerization was carried out for 48 h at 60°C. Ultrathin sections (70 nm) were cut using a Leica UC 6 ultramicrotome and mounted onto 200‐mesh copper grids. The grids with the sections were stained for 20 min with saturated uranyl acetate and subsequently stained for 10 min with lead citrate. The sections were imaged at 120 kV using a JEOL JEM‐1400plus (Tokyo, Japan) transmission electron microscope, and images were captured with a Gatan 2k × 2k digital camera (Gatan Inc.). Mitochondrial area (%) and individual mitochondrial size (μm^2^) were measured using ImageJ2 from two randomly selected 2000× images taken from a section of each muscle sample.

### 2.10 Skeletal Muscle Histology

To quantify muscle fibre cross‐sectional area (CSA), TA muscles were embedded in OCT compound and snap‐frozen in liquid nitrogen–cooled isopentane. Cryosections (10 μm) were prepared using a Leica CM1860 cryostat (Leica Biosystems). Sections were then incubated with wheat germ agglutinin (WGA) Alexa Fluor 488 conjugate (Thermo Fisher Scientific, W11261) at 4 μg/mL in PBS for 1 h, followed by three washes with PBS. After fixation in methanol for 5 min at room temperature and two PBS washes, sections were mounted with Antifade Mounting Medium (Vector Laboratories, H‐1000). Images were acquired using a Zeiss Axioscan 7 Slide Scanner (Carl Zeiss). Image analysis was performed using Cellpose v3.1 (Data S2: [S6, S7]) and ImageJ2 (Data S2: [S8]). Briefly, images were downsized to 50% (0.648 μm/pixel). Segmentation masks were generated using the Cellpose cyto3 model with specific parameters (diameter: 92, flow threshold: 2.5, probability threshold: 5). Fibre areas of the entire muscle section (3283 ± 74 fibres per section) were then quantified using ImageJ2, excluding fibres smaller than 600 μm^2^ as artefacts.

### 2.11 Ex Vivo Skeletal Muscle Force Production

The force production capacities of the EDL and SOL muscles were measured as previously described (Data S2: [S9]). Briefly, both the EDL and SOL muscles were secured at their tendons, then attached to an anchor and a force transducer within a tissue bath (Aurora Scientific, Model 801C) while being submerged in oxygenated Krebs–Henseleit buffer (KHB) at 37 C. The muscles were suspended at their optimal length, determined by pulse stimulation. Subsequently, the muscles were incubated in fresh KHB and allowed to equilibrate for 5 min. Following this equilibration period, a force–frequency protocol was initiated, stimulating the muscle at increasing frequencies (10, 20, 30, 40, 60, 80, 100, 125, 150 and 200 Hz) with a 2‐min rest interval between each frequency. Muscle length and mass were measured to estimate muscle CSA. Force production data were analysed using the Aurora Scientific DMAv5.321 software, and results were reported as specific forces that normalized absolute force to muscle CSA (Data S2: [S10]).

### 2.12 Statistical Analyses

Statistical analysis was performed using GraphPad Prism 10.1.1 software. Body mass and muscle mass were analysed separately for males and females. For analysis of all other data, both sexes were combined because no sex differences were observed in the values or the genotype effects. An unpaired Student's *t*‐test was used to compare differences between genotypes. Corrections for the false discovery rate were applied for the lipidomics data. For the mitochondrial bioenergetics, muscle force–frequency and muscle fibre CSA data, a two‐way analysis of variance (ANOVA) was conducted to explore the interaction between genotype and the other factors. If a significant interaction was observed, an appropriate post hoc multiple‐comparison test was subsequently applied. All data are represented as mean ± SEM and statistical significance was set at *p* ≤ 0.05.

## 3 Results

### 3.1 PGC‐1α Overexpression Alters Muscle Mitochondrial Lipidome

Wildtype and MCK‐PGC‐1α mice did not differ in body and muscle masses (Figure 1B,C). We confirmed that Pgc‐1α mRNA levels were increased in GAS muscles from MCK‐PGC‐1α mice compared with wildtype mice, and this was accompanied by increases in select markers of mitochondrial biogenesis (Figure 1D). Lipidomic analyses revealed a number of robust changes in the skeletal muscle mitochondrial lipidome. First, PGC‐1α overexpression promoted a 22% increase in lipid‐to‐protein ratio (Figure 1E), suggesting that PGC‐1α increases mitochondrial membrane lipid syntheses to a greater extent than mitochondrial proteins. PGC‐1α overexpression increased the concentrations of PE, CL and its precursor phosphatidylglycerol (PG), as well as lysophosphatidylcholine (LPC) and lysophosphatidylethanolamine (LPE) (Figure 1F). The purity of mitochondrial isolation in these samples was verified by Western blot (Figure S1A).

### 3.2 Skeletal Muscle PGC‐1α Overexpression Promotes PE and CL Biosynthesis in Isolated Mitochondria

**FIGURE 2:**
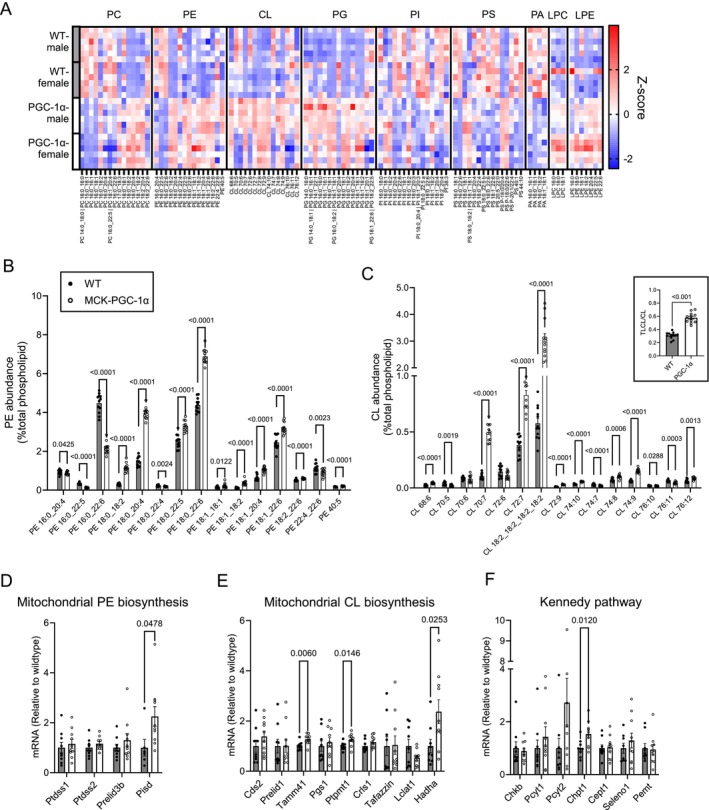
PGC‐1α promotes PE and CL biosynthesis in isolated muscle mitochondria. (A) Heatmap of the relative abundance of mitochondrial lipids. The X‐axis represents individual lipid species classified according to lipid classes (WT *n* = 12 [male:female = 6:6], MCK‐PGC‐1α *n* = 12 [male:female = 6:6]). (B) Abundance of individual phosphatidylethanolamine (PE) species in isolated mitochondria. (C) Abundance of individual cardiolipin (CL) species in isolated mitochondria. The ratio of tetralinoleoyl CL (TLCL) to total CL is presented as an insert. (D–F) mRNA levels of enzymes and transporters that relate to biosynthesis of mitochondrial PE (D) and CL (E), and Kennedy pathway (F) (WT *n* = 9 [male:female = 4:5], MCK‐PGC‐1α *n* = 10 [male:female = 5:5]). Data are represented as mean ± SEM.

By their relative abundance, PGC‐1α overexpression has the most robust effects to increase PE and CL concentrations in mitochondria compared with other lipid classes. For both PE and CL, however, the abundance of some species was reduced with PGC‐1α overexpression (Figures 2A and S1B). With respect to PE, PGC‐1α overexpression tended to reduce species containing 16‐carbon acyl chains in the SN1 position (Figure 2A,B). In the CL class, PGC‐1α led to a substantial increase in TLCL (tetralinoleoyl‐CL or 18:2/18:2/18:2/18:2‐CL, considered to be a functional form of CL) abundance, resulting in nearly a twofold higher ratio of TLCL to total CL compared with WT mice (Figure 2A,C). CL of low molecular weights also tended to be lower in female mice vs. male mice, regardless of genotype.

Because PGC‐1α altered PE and CL abundance, we examined whether PGC‐1α influenced the gene expression of enzymes involved in mitochondrial PE and CL biosynthesis. Mitochondrial PE is primarily generated from phosphatidylserine (PS) by the PS decarboxylase (Pisd) localized to the IMM, which was increased by PGC‐1α overexpression (Figure 2D). CL is almost exclusively synthesized by a series of enzymes localized in the IMM that convert PA into PG, PG into CL and convert generic CL into TLCL. PGC‐1α increased or tended to increase mRNA levels for some of these enzymes (Figure 2E). There were additional modest and nuanced changes in the mRNA levels for enzymes in the endoplasmic reticulum phospholipid biosynthesis (Kennedy pathway) that supply substrates for mitochondrial lipid biosynthesis (Figure 2F).

### 3.3 PGC‐1α Overexpression Enhances Respiratory Capacity but Does Not Change OXPHOS Efficiency in PmFB

**FIGURE 3:**
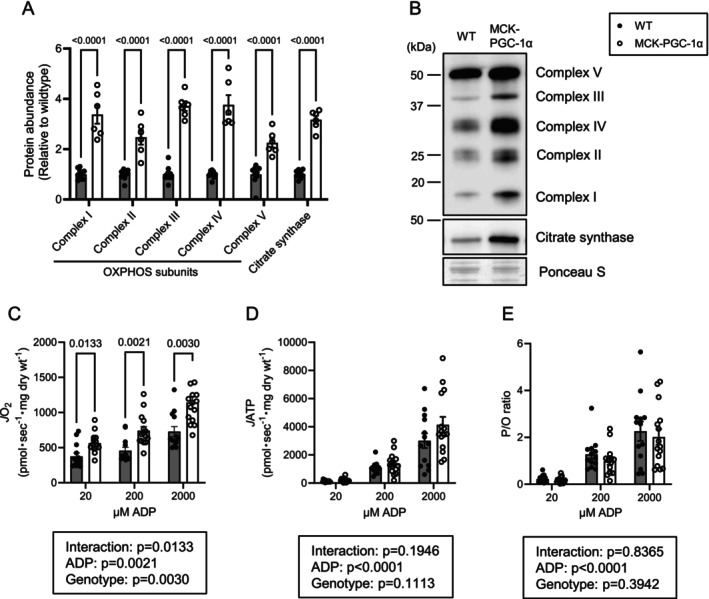
PGC‐1α overexpression does not change OXPHOS energy efficiency in PmFB. (A) Abundance of OXPHOS subunits and citrate synthase in the whole lysate of gastrocnemius muscle (WT *n* = 10 [male:female = 5:5], MCK‐PGC‐1α *n* = 6 [male:female = 3:3]). (B) Representative Western blots of OXPHOS subunits and citrate synthase. (C–E) Rate of ATP production (C), rate of oxygen consumption (D) and P/O ratio (E) in fibre bundles isolated from red gastrocnemius muscle (WT *n* = 13 [male:female = 9:4], MCK‐PGC‐1α *n* = 15 [male:female = 9:6]). Data are represented as mean ± SEM.

Consistent with previous reports [14, 17], PGC‐1α overexpression increased the abundance of OXPHOS subunits and citrate synthase in whole muscle lysates (Figure 3A,B). High‐resolution respirometry demonstrated that PGC‐1α overexpression increases O_2_ consumption (*J*O_2_) in permeabilized fibres (PmFB) (Figure 3C). PGC‐1α also tended to increase the ATP production rate (*J*ATP), although to a lesser extent than *J*O_2_ (Figure 3D). Together, PGC‐1α did not alter P/O in skeletal muscle PmFB (the mean was lower, not higher, in MCK‐PGC‐1α compared with wildtype) (Figure 3E).

### 3.4 PGC‐1α Overexpression Does Not Robustly Alter the OXPHOS Efficiency in Isolated Muscle Mitochondria

**FIGURE 4:**
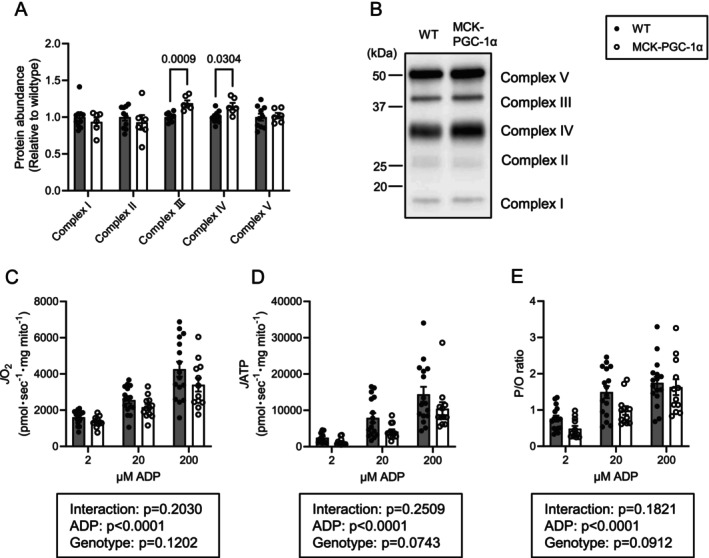
PGC‐1α overexpression does not alter OXPHOS energy efficiency in isolated muscle mitochondria. (A) Abundance of OXPHOS subunits in isolated mitochondria from gastrocnemius muscle (WT *n* = 10 [male:female = 5:5], MCK‐PGC‐1α *n* = 6 [male:female = 3:3]). (B) Representative western blots of OXPHOS subunits. (C–E) Rate of ATP production (C), rate of oxygen consumption (D) and P/O ratio (E) in isolated mitochondria from gastrocnemius muscle (WT *n* = 16 [male:female = 8:8], MCK‐PGC‐1α *n* = 12 [male:female = 6:6]). Data are represented as mean ± SEM.

While studying PmFB allows us to examine mitochondria in an ecosystem that is more similar to the in vivo environment, the preparation procedures also yield organelles and enzyme systems that may interfere with mitochondrial metabolism in situ. Thus, to further study the influence of PGC‐1α on OXPHOS efficiency, we performed the same set of experiments also in isolated muscle mitochondria. Because units are normalized to the abundance of mitochondrial proteins, the effect of PGC‐1α to increase OXPHOS subunits (Figure 3A,B) largely went away except for modest increases in subunits for Complexes III and IV (Figure 4A,B). Contrary to our hypothesis, PGC‐1α overexpression tended to reduce *J*O_2_, *J*ATP and P/O in skeletal muscle mitochondria (Figure 4C–E).

### 3.5 PGC‐1α Overexpression Promotes Mitochondrial Remodelling and Supercomplex Assembly

**FIGURE 5:**
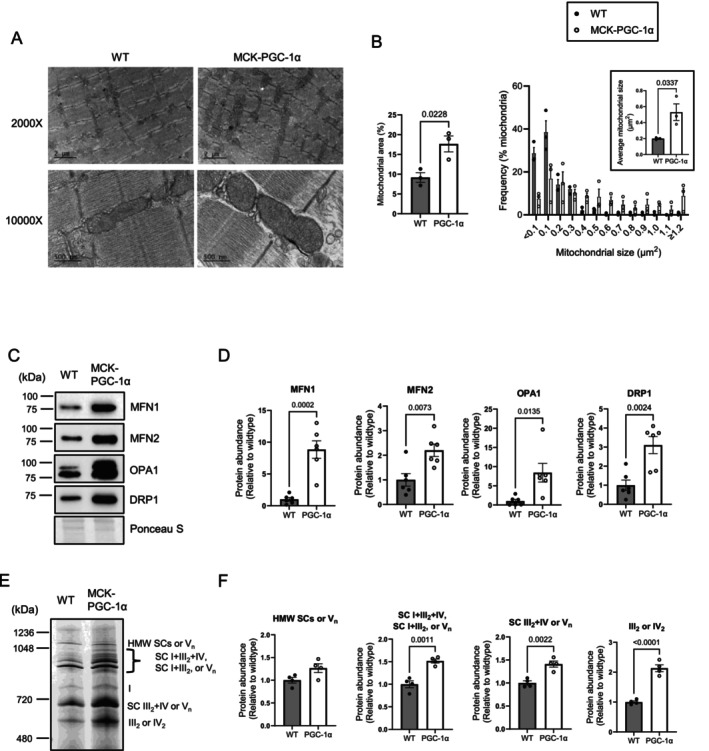
PGC‐1α overexpression promotes mitochondrial remodelling and supercomplex formation. (A) Representative electron microscopy images of gastrocnemius muscle. (B) Quantifications of mitochondrial area and individual mitochondrial size (WT *n* = 3 [male], MCK‐PGC‐1α *n* = 3 [male]). (C) Representative Western blots of mitochondrial dynamics proteins in whole lysate of gastrocnemius muscle. (D) Abundance of mitochondrial dynamics proteins (WT *n* = 6 [male:female = 3:3], MCK‐PGC‐1α *n* = 6 [male:female = 3:3]). (E) Representative image of mitochondrial supercomplexes in isolated mitochondria from gastrocnemius muscle. (F) Abundance of mitochondrial supercomplexes (WT *n* = 4 [male:female = 2:2], MCK‐PGC‐1α *n* = 4 [male:female = 2:2]). Data are represented as mean ± SEM.

Our findings that PGC‐1α overexpression did not alter mitochondrial P/O led us to further evaluate properties of mitochondria that have been implicated in influencing efficiency. We first examined the influence of PGC‐1α overexpression on mitochondrial morphology. Electron micrographs strikingly showed an increase in mitochondrial area, with enlargement of individual mitochondrial size in MCK‐PGC‐1α mice compared with wildtype controls (Figure 5A top and 5B). Aligning with these findings, Western blotting revealed a more pronounced increase in mitochondrial fusion proteins, including MFN1 and OPA1, compared with the mitochondrial fission protein DRP1 in MCK‐PGC‐1α mice (Figure 5C,D). These proteins have also been implicated in cristae formation, but we did not see robust changes in cristae morphology with PGC‐1α overexpression (Figure 5A, bottom). Supercomplex assembly has been implicated in the efficient transfer of electrons in the electron transport chain and can increase with exercise [29]. Indeed, we saw a striking increase in essentially all forms of respiratory supercomplexes (Figure 5D,E).

### 3.6 Skeletal Muscle PGC‐1α Overexpression Reduces Force‐Generating Capacity

**FIGURE 6:**
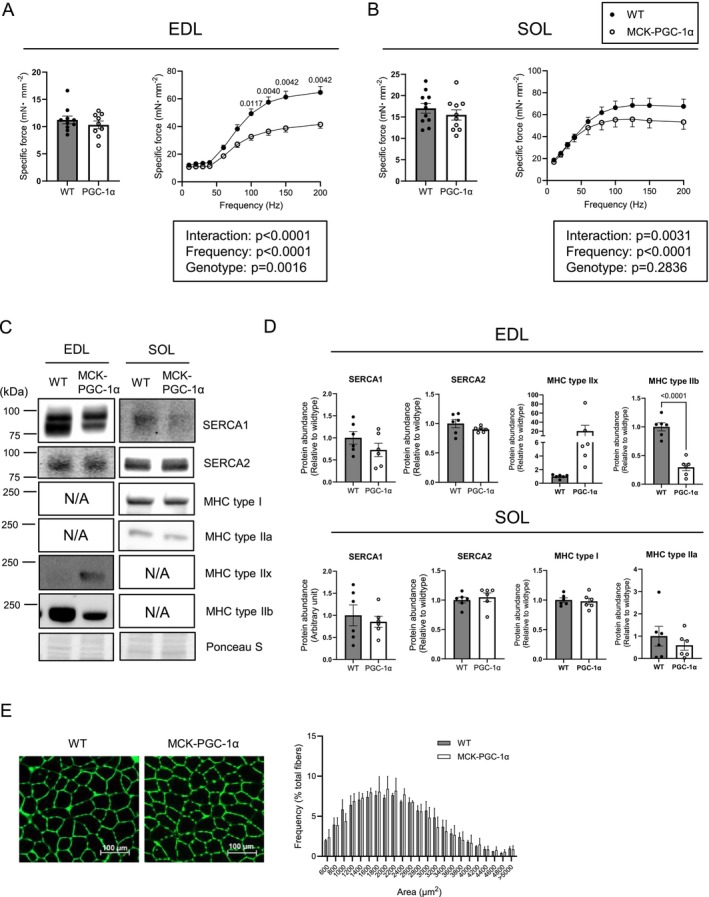
Overexpression of PGC‐1α reduces muscle force production in EDL muscle. (A) Force produced with a pulse stimulation and tetanic stimulation ranging from 10 to 200 Hz in extensor digitorum longus (EDL) muscle (WT *n* = 10 [male:female = 5:5], MCK‐PGC‐1α *n* = 9 [male:female = 4:5]). (B) Force produced with a pulse stimulation and tetanic stimulation ranging from 10 to 200 Hz in soleus (SOL) muscle (WT *n* = 11 [male:female = 5:6], MCK‐PGC‐1α *n* = 10 [male:female = 5:5]). (C) Representative Western blots of SERCA proteins and myosin heavy chain (MHC) isoforms in whole lysate of EDL and SOL muscle (WT *n* = 6 [male:female = 3:3], MCK‐PGC‐1α *n* = 6 [male:female = 3:3]). (D) Abundance of SERCA proteins and MHC isoforms in EDL and SOL muscle. (E) Representative tibialis anterior (TA) muscle sections stained with wheat germ agglutinin and quantification of muscle fibre cross‐sectional area (WT *n* = 3 [male], MCK‐PGC‐1α *n* = 3 [male]). Data are represented as mean ± SEM.

Prompted by trends for reduced *J*ATP (*p* = 0.0743) and P/O (*p* = 0.0912), we examined a potential functional consequence of such a bioenergetic shift by PGC‐1α overexpression. Surprisingly, but consistent with the results from high‐resolution fluorometry and respirometry, PGC‐1α overexpression reduced the force‐generating capacity of fast‐twitch EDL muscle (Figure 6A), although the reduction was less pronounced in the slow‐twitch SOL muscle (Figure 6B). We further examined the isoforms of SERCA and myosin ATPases, as changes in these isoforms could contribute to changes in force‐generating capacity. While previous studies have suggested that PGC‐1α overexpression leads to a reduction in calcium‐handling proteins [17, 30], we did not observe robust changes in SERCA1 or SERCA2 in either EDL or SOL muscles (Figure 6C,D). In contrast, PGC‐1α overexpression promoted a drastic shift in MHC isoform from type IIb to IIx in the fast‐twitch dominant EDL and TA muscles, but did not induce any fibre type shift in the slow‐twitch dominant SOL muscle (Figures 6C,D and S3), suggesting these changes might be responsible for the lower force‐generating capacity in MCK‐PGC‐1α vs. wildtype mice [31, 32]. There was no significant difference in muscle fibre CSA in the TA muscle between genotypes (Figure 6E).

## 4 Discussion

Despite extensive research on the mechanisms regulating mitochondrial biogenesis and respiration [6, 7], studies on the regulation of energy efficiency for ATP synthesis are limited. This study was conducted to test the hypothesis that PGC‐1α remodels skeletal muscle mitochondrial membrane lipid composition, which in turn may induce an increase in OXPHOS efficiency. PGC‐1α overexpression indeed induced remodelling of the mitochondrial membrane lipids, particularly for the biosynthesis of mitochondrial PE and CL. However, PGC‐1α overexpression alone was insufficient to increase mitochondrial P/O. PGC‐1α overexpression increased the formation of respiratory supercomplexes as well as proteins involved in mitochondrial fusion and fission, suggesting that these mechanisms may be insufficient to influence the efficiency of ATP synthesis.

Some studies in rodents and humans have demonstrated that efficiency for mitochondrial OXPHOS is altered in response to exercise training and aging. Skeletal muscle mitochondria from rats subjected to 8 weeks of running exercise training demonstrated higher OXPHOS energetic efficiency compared with sedentary rats [2]. In humans, 6 weeks of high‐intensity training was found to improve skeletal muscle mitochondrial OXPHOS efficiency concomitant with greater performance efficiency [3]. In contrast, the P/O values in the skeletal muscle from the aged mice (30‐month‐old) were shown to be lower than those found in the muscle of young adult mice (7‐month‐old) [4]. In humans, exercise efficiency measured during a submaximal cycle test was lower in older adults (71 ± 4) than in younger adults (24 ± 3) [33]. However, these findings are not universal, as other studies demonstrate no change in OXPHOS efficiency with exercise training or aging [34, 35]. The reason for this discrepancy is unclear, but it is likely due to differences in dose, intensity, mode, frequency and duration of exercise training, under the background of subjects with different age, sex and race. We initially set out to study MCK‐PGC‐1α mice partly because it is thought to exhibit robust skeletal muscle mitochondrial adaptations, presumably reliably phenocopying subsets of mitochondrial properties induced by exercise training. In retrospect, the lack of change in P/O with PGC‐1α overexpression in mice does not preclude whether exercise training or aging does or does not increase muscle efficiency for ATP synthesis. Particularly in the context of preclinical studies on exercise adaptation, rats have historically been used as the preferred model, and there is a great paucity in the types of exercise training protocols in mice that would induce adaptations like those in humans. One might say that it is yet unknown whether exercise, aging or PGC‐1α overexpression influences the efficiency of skeletal muscle ATP synthesis in mice.

We have previously shown that metabolic challenges, such as exercise training or weight loss, remodel the lipid composition of mitochondrial membranes, and that such changes can influence mitochondrial respiratory enzymes and/or P/O [8, 25]. Given the well‐known role of PGC‐1α in mediating many of the mitochondrial adaptations [10, 11, 36], we initially hypothesized that PGC‐1α also remodels skeletal muscle mitochondrial membrane lipid composition. Indeed, PGC‐1α overexpression promoted robust changes in mitochondrial membrane lipid composition, including increased PE and CL, changes that resembled those occurring with exercise training as well as differences observed between high‐capacity running rats and low‐capacity running rats [23]. PGC‐1α overexpression also increased the proportion of TLCL as seen in skeletal muscle experiencing overload or weight loss [8, 37]. Consistent with these findings, we observed increases in mRNA of enzymes that regulate PE and CL biosynthesis in skeletal muscle with PGC‐1α overexpression. These findings are aligned with the notion that PGC‐1α mediates the mitochondrial membrane lipid modelling. The exact nature in which these changes facilitate bioenergetic adaptations would require additional studies.

Fuel preference in muscles from MCK‐PGC‐1α mice shifts to fatty acid substrate rather than carbohydrate substrate compared with wildtype controls [16, 38, 39]. The switch in substrate preference that occurs with PGC‐1α overexpression or those that occur with exercise training is likely a direct result of increased mitochondrial density, whereby a shift in dynamics for high‐energy phosphates allosterically influences substrate‐processing enzymes in a way that promotes fatty acid catabolism over glucose catabolism [40]. Carbohydrates and lipids are known to have different yields for ATP per oxygen molecule, and one might predict that the difference in fuel preference might influence P/O. Nevertheless, it is in fact glucose, rather than fatty acids, that yields a greater amount of ATP per oxygen molecule. It remains possible that palmitate‐induced P/O may differ between wildtype and MCK‐PGC‐1α mice.

MCK‐PGC‐1α overexpression decreased maximal force in the EDL muscles. This study is not the first to report that PGC‐1α overexpression attenuated muscle force production [17, 30]. Summermatter et al. suggested that overexpression of PGC‐1α decreases calcium release, contributing to reduced maximal force [30]. In the current study, we did not observe significant differences in SERCA protein abundance in either EDL or SOL muscles. Instead, as previously reported [31, 32], PGC‐1α overexpression promoted a shift from MHC IIb to IIx, shifting towards a more oxidative MHC isoform in fast‐twitch EDL and TA muscle. As MHC IIb fibres demonstrate greater force‐generating capacity, these results likely explain the reduced ex vivo force production observed in the EDL muscle of MCK‐PGC‐1α mice compared with wildtype mice. On a related note, previous studies have reported no differences in the P/O ratio between fast‐twitch glycolytic and slow‐twitch oxidative muscle (Data S2: [S11, S12]). It is also important to note that our experiments with P/O included EGTA and blebbistatin that would prevent SERCA‐ and MHC‐dependent ATP consumption, respectively. Thus, the fibre‐type shift induced by PGC‐1α overexpression is unlikely to influence OXPHOS efficiency.

Although evaluating mitochondrial bioenergetics using both PmFB and isolated mitochondria represents a major strength of this study, it is important to acknowledge the methodological limitations of each method. The preparation of PmFB retains organelles and enzymatic systems that may interfere with mitochondrial metabolism in situ. It has also been reported that potential contraction may influence assay outcome (Data S2: [S13]), although we include blebbistatin to minimize this effect. On the other hand, the process of mitochondrial isolation has been reported to compromise certain physiological functions, such as sensitivity to Ca^2+^‐induced opening of the permeability transition pore, H_2_O_2_‐emitting potential and the stoichiometry between ATP synthase and complex I (Data S2: [S14]). Nevertheless, the consistent lack of genotype differences in our primary outcome measure, the P/O, across both assays supports the conclusion that PGC‐1α overexpression does not enhance OXPHOS efficiency.

In conclusion, the current study demonstrates that overexpression of PGC‐1α does not alter OXPHOS efficiency in mouse skeletal muscle. While overexpression of PGC‐1α promoted the biosynthesis of mitochondrial PE and CL, these changes were insufficient to increase P/O. The findings suggest that PGC‐1α is sufficient to promote increases in mitochondrial PE and CL, suggesting that PGC‐1α‐dependent mechanisms may drive the changes in mitochondrial membrane lipids induced by exercise training. There remains a possibility that these mechanisms synergize with PGC‐1α‐independent mechanisms to influence skeletal muscle mitochondrial P/O.

## Conflicts of Interest

The authors declare no conflicts of interest.

## Supporting information


**Data S1:** Supporting information.

**Figure S1:** Overexpression of PGC‐1α remodels mitochondrial phospholipid composition in skeletal muscle. (A) Validation of mitochondrial isolation purity by Western blot using the cytoplasmic marker GAPDH and the mitochondrial marker COX IV (WT *n* = 3 [male], MCK‐PGC‐1α *n* = 3 [male]). (B) Abundance of individual lipid species of phosphatidylcholine (PC), phosphatidylglycerol (PG), phosphatidylinositol (PI), phosphatidylserine (PS), phosphatidic acid (PA), lysophosphatidylcholine (LPC) and lysophosphatidylethanolamine (LPE) in isolated muscle mitochondria (WT *n* = 12 [male:female = 6:6], MCK‐PGC‐1α *n* = 12 [male:female = 6:6]). Data are represented as mean ± SEM.
**Figure S2:** Overexpression of PGC‐1α does not alter OXPHOS efficiency in PmFB or isolated mitochondria, regardless of sex. (A) Rate of ATP production, rate of oxygen consumption and P/O ratio in PmFB from male mice (WT = 9, MCK‐PGC‐1α = 9). (B) Rate of ATP production, rate of oxygen consumption and P/O ratio in PmFB from female mice (WT = 4, MCK‐PGC‐1α = 6). (C) Rate of ATP production, rate of oxygen consumption and P/O ratio in isolated muscle mitochondria from male mice (WT = 8, MCK‐PGC‐1α = 6). (D) Rate of ATP production, rate of oxygen consumption and P/O ratio in isolated muscle mitochondria from female mice (WT = 8, MCK‐PGC‐1α = 6). Data are represented as mean ± SEM.
**Figure S3:** PGC‐1α overexpression promoted a shift in myosin heavy chain isoform from type IIb to IIx in the TA muscle. (A) Abundance of myosin heavy chain isoforms (MHC) in tibialis anterior (TA) muscle (WT *n* = 6 [male:female = 3:3], MCK‐PGC‐1α *n* = 6 [male:female = 3:3]). (B) Representative Western blots of MHC isoforms. Data are represented as mean ± SEM.

**Table S1:** Primers for quantitative PCR.

**Data S2:** Supporting information.

**Data S3:** Supporting information.
