# Supplementary figures and images for: Skeletal Muscle PGC‐1α Remodels Mitochondrial Phospholipidome but Does Not Alter Energy Efficiency for ATP Synthesis
Source: J Cachexia Sarcopenia Muscle. 2025 Oct 9;16(5):e70090. doi: 10.1002/jcsm.70090 (PMC12511762; doi:10.1002/jcsm.70090)

Figure S1

A

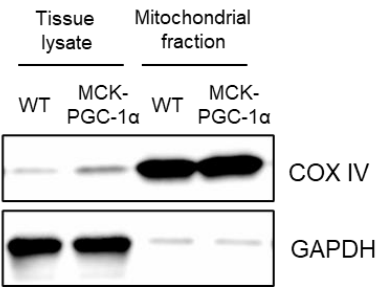

B

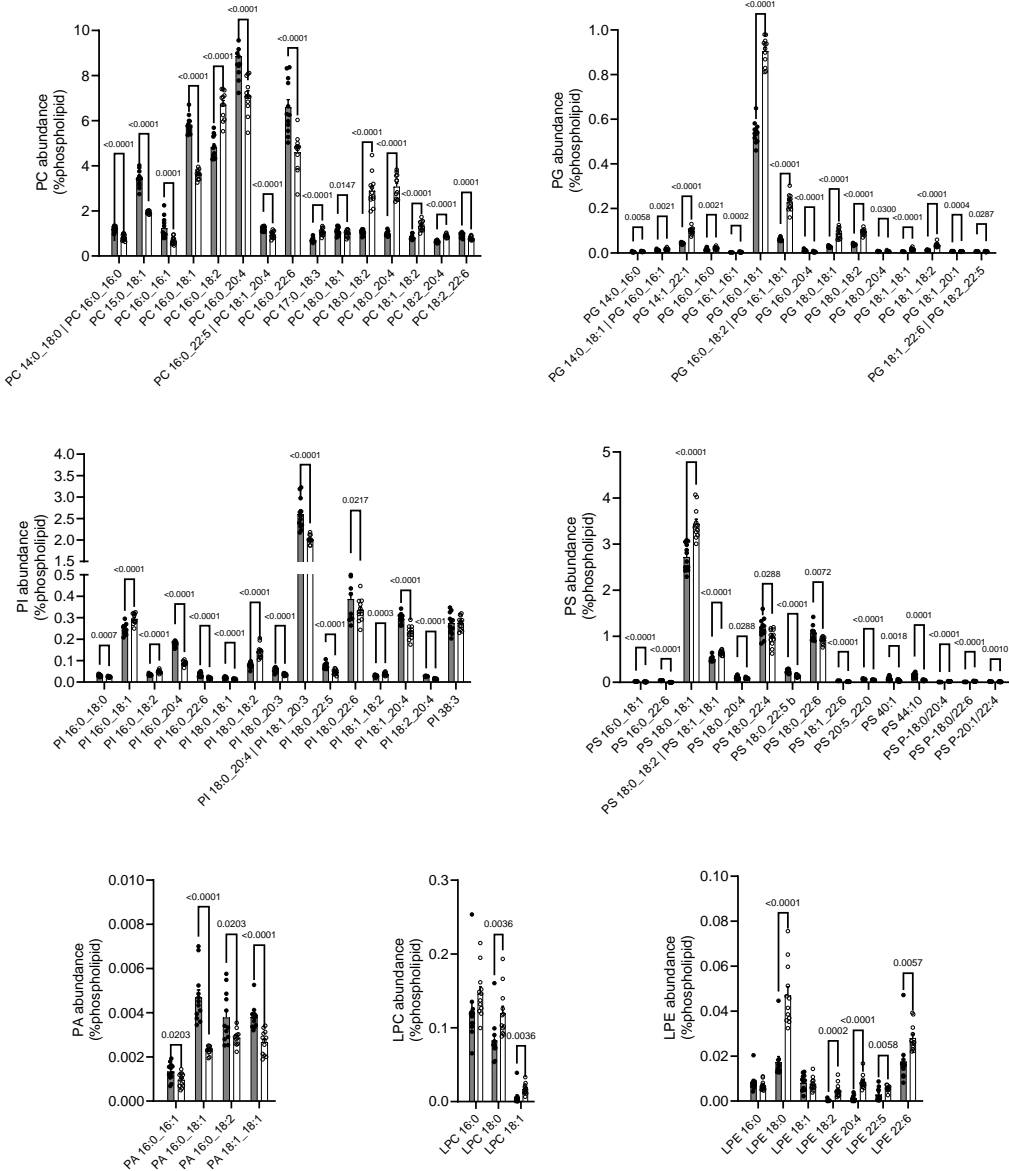

# Figure S2

## Permeabilized fiber bundle

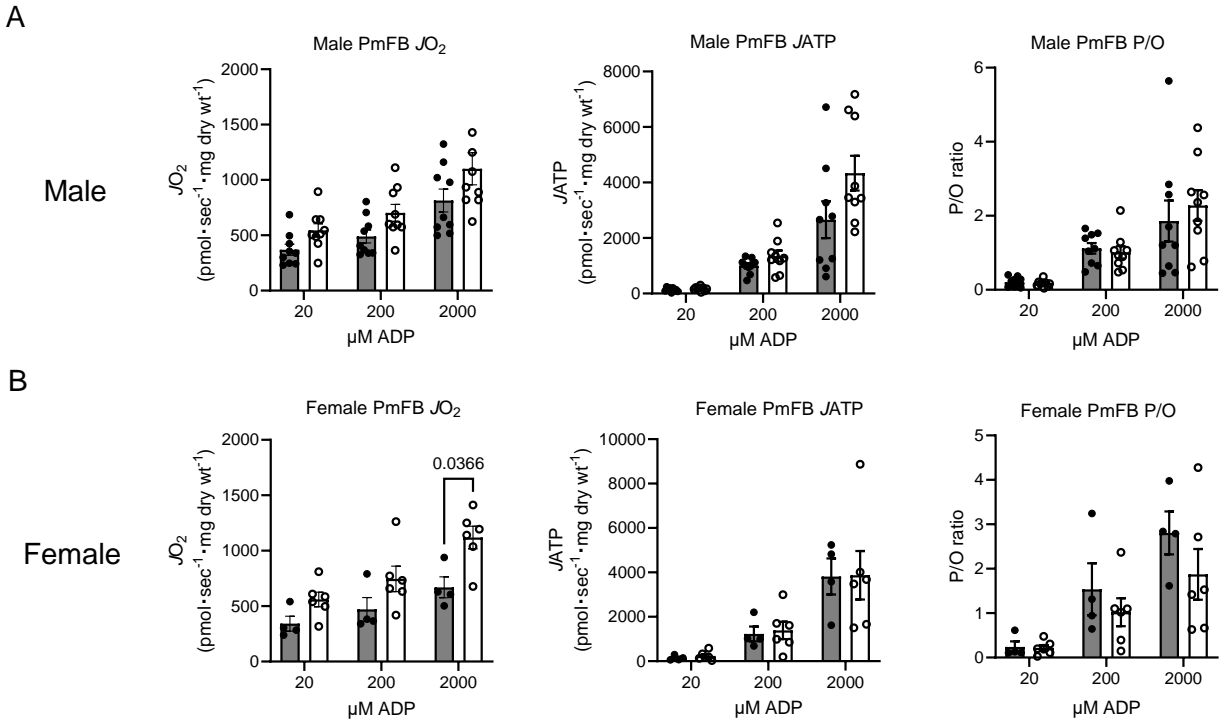

## Isolated mitochondria

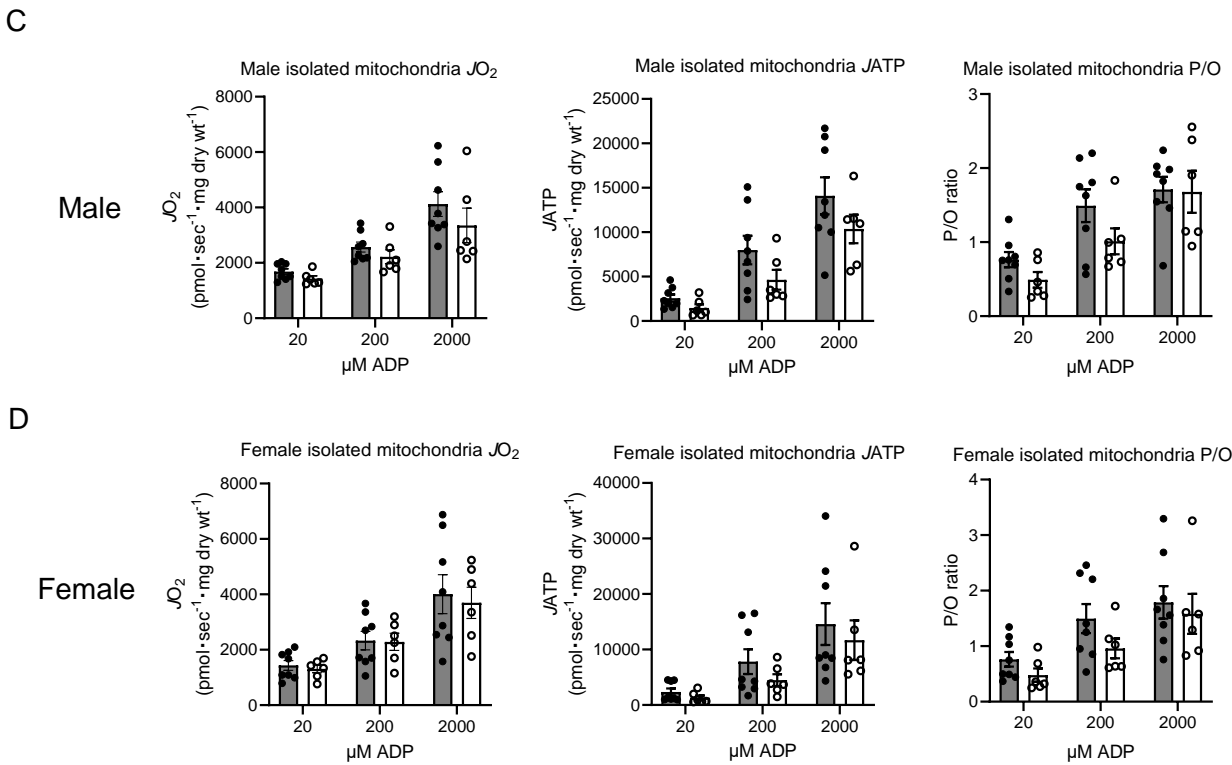

Figure S3

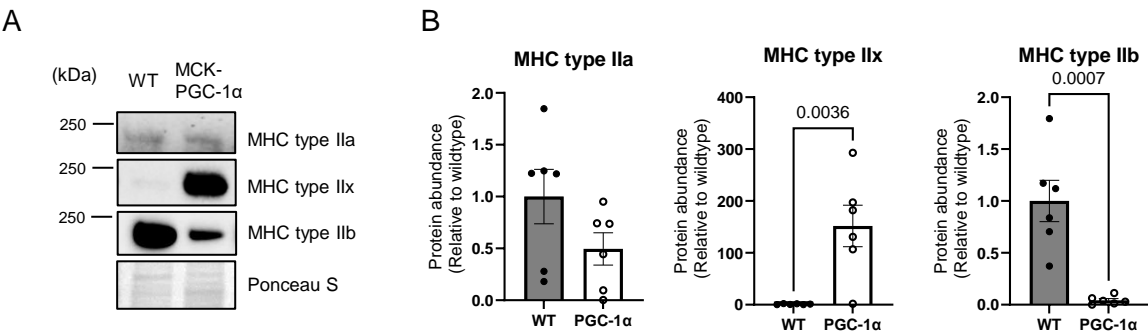

Supplement: Supplementary file 1 — Data S1: Supporting information. [file JCSM-16-e70090-s003.pdf]

### Figure 3B

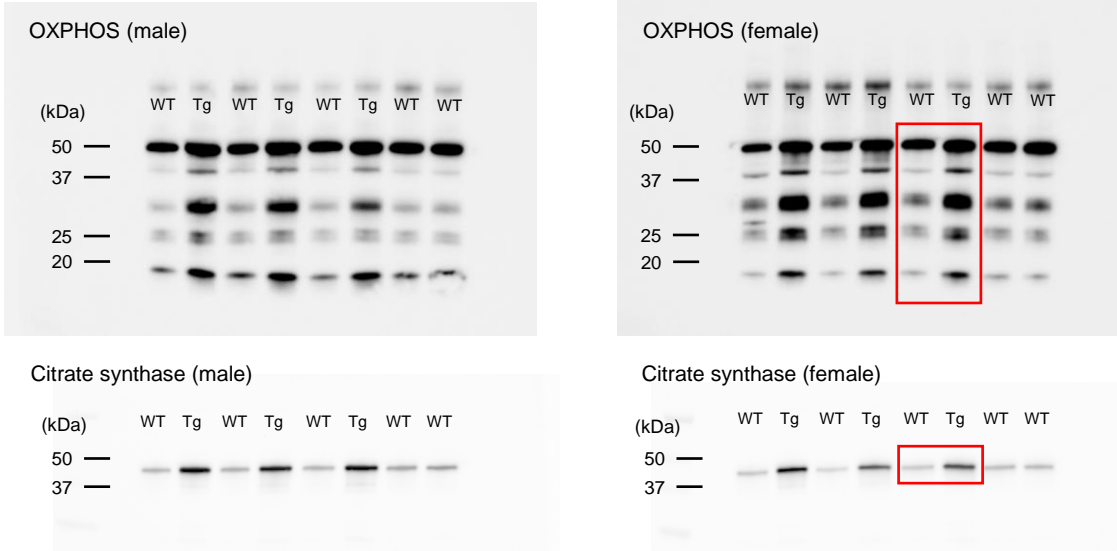

### Figure 4B

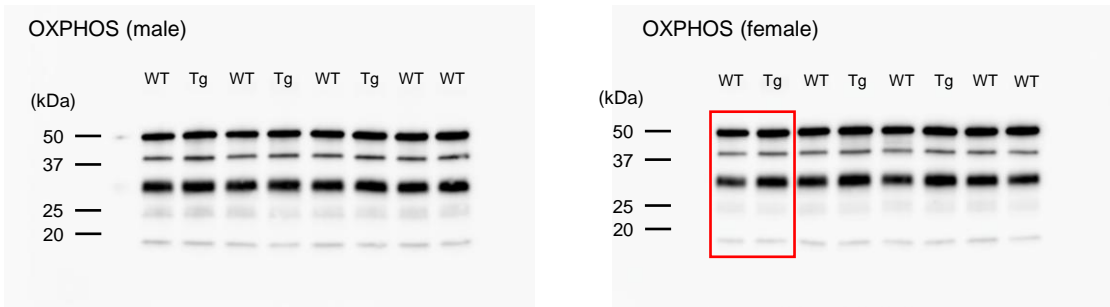

### Figure 5B

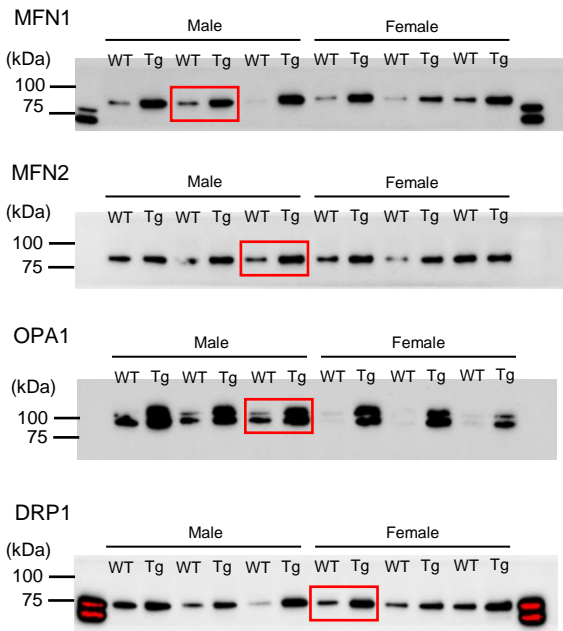

### Figure 5D

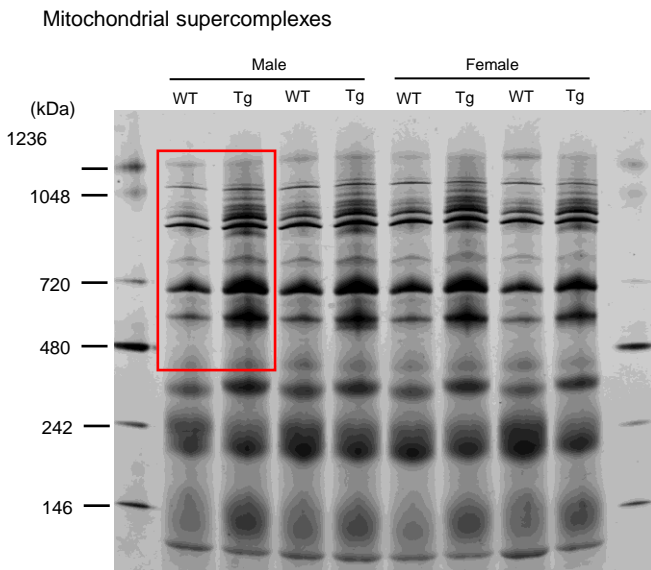

Figure 6C

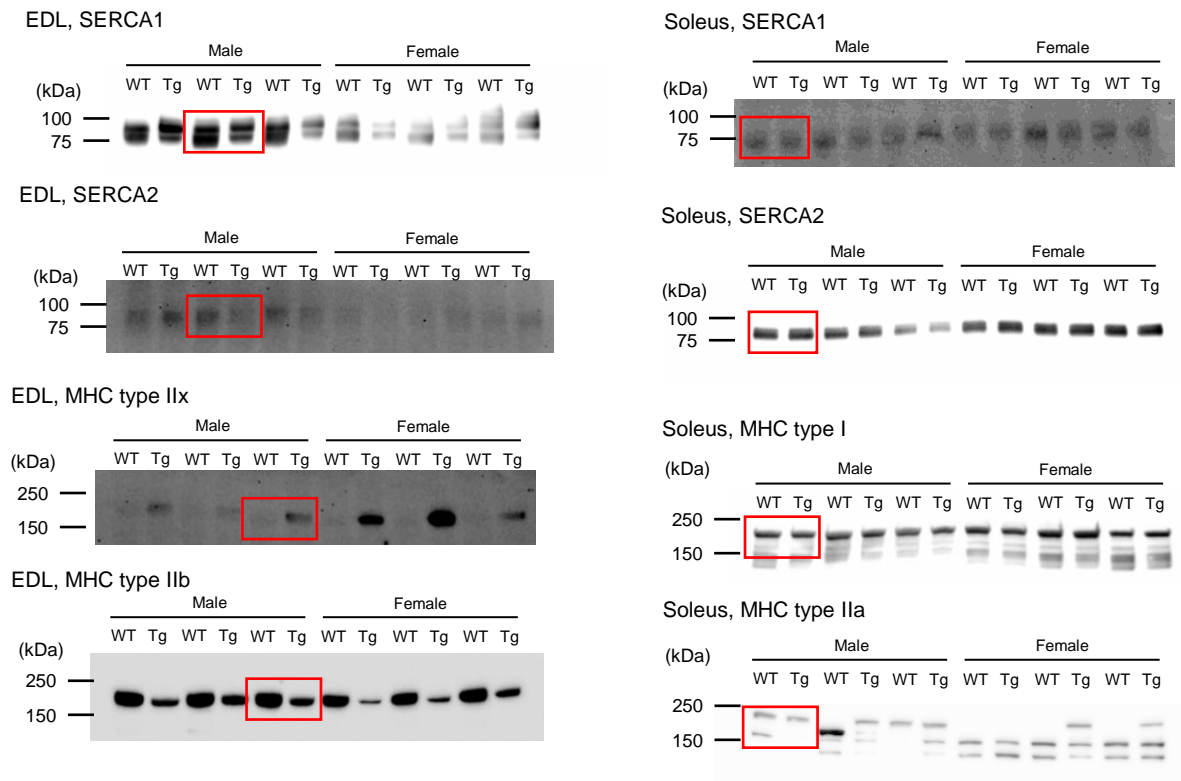

Figure S3B

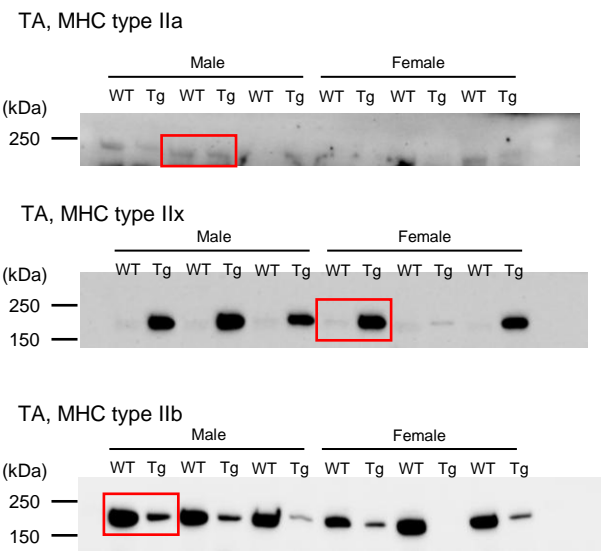

Supplement: Supplementary file 5 — Data S3: Supporting information. [file JCSM-16-e70090-s005.pdf]
