# Supplementary material for: Skeletal Muscle PGC‐1α Remodels Mitochondrial Phospholipidome but Does Not Alter Energy Efficiency for ATP Synthesis
Source: J Cachexia Sarcopenia Muscle. 2025 Oct 9;16(5):e70090. doi: 10.1002/jcsm.70090 (PMC12511762; doi:10.1002/jcsm.70090)
Supplement: Supplementary file 2 — Figure S1: Overexpression of PGC‐1α remodels mitochondrial phospholipid composition in skeletal muscle. (A) Validation of mitochondrial isolation purity by Western blot using the cytoplasmic marker GAPDH and the mitochondrial marker COX IV (WT n = 3 [male], MCK‐PGC‐1α n = 3 [male]). (B) Abundance of individual lipid species of phosphatidylcholine (PC), phosphatidylglycerol (PG), phosphatidylinositol (PI), phosphatidylserine (PS), phosphatidic acid (PA), lysophosphatidylcholine (LPC) and lysophosphatidylethanolamine (LPE) in isolated muscle mitochondria (WT n = 12 [male:female = 6:6], MCK‐PGC‐1α n = 12 [male:female = 6:6]). Data are represented as mean ± SEM. Figure S2: Overexpression of PGC‐1α does not alter OXPHOS efficiency in PmFB or isolated mitochondria, regardless of sex. (A) Rate of ATP production, rate of oxygen consumption and P/O ratio in PmFB from male mice (WT = 9, MCK‐PGC‐1α = 9). (B) Rate of ATP production, rate of oxygen consumption and P/O ratio in PmFB from female mice (WT = 4, MCK‐PGC‐1α = 6). (C) Rate of ATP production, rate of oxygen consumption and P/O ratio in isolated muscle mitochondria from male mice (WT = 8, MCK‐PGC‐1α = 6). (D) Rate of ATP production, rate of oxygen consumption and P/O ratio in isolated muscle mitochondria from female mice (WT = 8, MCK‐PGC‐1α = 6). Data are represented as mean ± SEM. Figure S3: PGC‐1α overexpression promoted a shift in myosin heavy chain isoform from type IIb to IIx in the TA muscle. (A) Abundance of myosin heavy chain isoforms (MHC) in tibialis anterior (TA) muscle (WT n = 6 [male:female = 3:3], MCK‐PGC‐1α n = 6 [male:female = 3:3]). (B) Representative Western blots of MHC isoforms. Data are represented as mean ± SEM. [file JCSM-16-e70090-s002.docx]

**Fig. S1 Overexpression of PGC-1α remodels mitochondrial phospholipid composition in skeletal muscle** (A) Validation of mitochondrial isolation purity by Western blot using the cytoplasmic marker GAPDH and the mitochondrial marker COX IV [WT n = 3 (male), MCK-PGC-1α n =3 (male)]. (B) Abundance of individual lipid species of phosphatidylcholine (PC), phosphatidylglycerol (PG), phosphatidylinositol (PI), phosphatidylserine (PS), phosphatidic acid (PA), lysophosphatidylcholine (LPC), and lysophosphatidylethanolamine (LPE) in isolated muscle mitochondria [WT n = 12 (male:female = 6:6), MCK-PGC-1α n = 12 (male:female = 6:6)]. Data are represented as mean ± SEM.

**Fig. S2 Overexpression of PGC-1α does not alter OXPHOS efficiency in PmFB or isolated mitochondria, regardless of sex** (A) Rate of ATP production, rate of oxygen consumption, and P/O ratio in PmFB from male mice (WT = 9, MCK-PGC-1α = 9). (B) Rate of ATP production, rate of oxygen consumption, and P/O ratio in PmFB from female mice (WT = 4, MCK-PGC-1α = 6). (C) Rate of ATP production, rate of oxygen consumption, and P/O ratio in isolated muscle mitochondria from male mice (WT = 8, MCK-PGC-1α = 6). (D) Rate of ATP production, rate of oxygen consumption, and P/O ratio in isolated muscle mitochondria from female mice (WT = 8, MCK-PGC-1α = 6). Data are represented as mean ± SEM.

**Fig. S3 PGC-1α overexpression promoted a shift in myosin heaviy chain isoform from type IIb to IIx in the TA muscle** (A) Abundance of myosin heaviy chain isoforms (MHC) in tibialis anterior (TA) muscle [WT n = 6 (male:female = 3:3), MCK-PGC-1α n = 6 (male:female = 3:3)]. (B) Representative Western blots of MHC isoforms. Data are represented as mean ± SEM.
