# Supplementary material for: Skeletal Muscle PGC‐1α Remodels Mitochondrial Phospholipidome but Does Not Alter Energy Efficiency for ATP Synthesis
Source: J Cachexia Sarcopenia Muscle. 2025 Oct 9;16(5):e70090. doi: 10.1002/jcsm.70090 (PMC12511762; doi:10.1002/jcsm.70090)
Supplement: Supplementary file 3 — Table S1: Primers for quantitative PCR. [file JCSM-16-e70090-s004.docx]

| Gene symbol | Forward (5’–3’) | Reverse (5’–3’) |
| --- | --- | --- |
| Pgc-1a | TGAGGACCGCTAGCAAGTTT | TGAAGTGGTGTAGCGACCAA |
| Tfam | CCAAAAAGACCTCGTTCAGC | TCCAAGCCTCATTTACAAGC |
| Cox4i1 | GAGCCTGATTGGCAAGAGAG | GATCAGCGTAAGTGGGGAAA |
| Chkb | GCAGAGGTTCAGAAGGGTGA | CCCCAGAAAAAGTGAGATGC |
| Pcyt1 | GGCATGACCAGAGTGAAACA | AGCCCTATGTCAGGGTGACT |
| Pcyt2 | AGGAGAGGTACAAGATGGTACAGG | GCTTCACTTCCTCATAGGTGTCTC |
| Chpt1 | CATCAACCTGGTCACCACAC | CCCAGGGCACATAAAAGGTA |
| Cept1 | TTCTGGAACATTGCGATTTG | AAAAGGTGGTCCTCCAATCA |
| Seleno1 | GCTTTGGATACCAACCCACTCTC | GGTCGAAGTATGTCAGGAGTAGG |
| Pemt | TCCTCAAGGAGTCCAGAGTGAC | ATTGCCACCAGCACCGTCAACA |
| Ptdss1 | ATCACCCTGCTCAGCTTCAC | CAGGATGCCTCTCCAGATGT |
| Ptdss2 | AAACCCCTCAGGATACAGCC | GGAAAATGGCCCGTCTTTAG |
| Prelid3b | GGACTTCGGAGCACGTCTTT | AACTTTCCAGAGGGATCGACA |
| Pisd | GTACAGGGAACGGAAGCTTGA | CCGGAGCCAGTAAGGAAGTT |
| Cds2 | GCTAGATGGAGAGACAGCGT | CGATCATGGCCAAAGTCAGG |
| Prelid1 | CATGACCACCTTCACCTGGAAC | GGATTTCGGTCCAGCCGCTATT |
| Tamm41 | AGACGTGGAAGAGACTCTGCTG | TCTGCCTGATGCTGGAAGGTCT |
| Pgs1 | GAAGTTTCCTTCCGACCTCAAG | AGCAGCATAGTGCGAGAGTTC |
| Ptpmt1 | CTATGAACGAGGAGTACGAGACC | AACTGGACTCCTTTGTGGAGATT |
| Crls1 | TGACCTATGCAGATCTTATTCCA | TGGCAGAGTTCGGTATCTGA |
| Taz | CCCTCCATGTGAAGTGGCCATTCC | TGGTGGTTGGAGACGGTGATAAGG |
| Alcat1 | ATTTTGCTGAGAAGAACGGACTT | TCCACCACAAAGGTAAAGCCA |
| Hadha | TGCATTTGCCGCAGCTTTAC | GTTGGCCCAGATTTCGTTCA |
| mtDNA | TTAAGACACCTTGCCTAGCCACAC | CGGTGGCTGGCACGAAATT |

Table S1. Primers for quantitative PCR
