# Supplementary material for: Skeletal Muscle PGC‐1α Remodels Mitochondrial Phospholipidome but Does Not Alter Energy Efficiency for ATP Synthesis
Source: J Cachexia Sarcopenia Muscle. 2025 Oct 9;16(5):e70090. doi: 10.1002/jcsm.70090 (PMC12511762; doi:10.1002/jcsm.70090)
Supplement: Supplementary file 4 — Data S2: Supporting information. [file JCSM-16-e70090-s001.docx]

**Supplemental References**

S1. Matyash V, Liebisch G, Kurzchalia TV, Shevchenko A, Schwudke D. Lipid extraction by methyl-tert-butyl ether for high-throughput lipidomics. J Lipid Res. 2008;49:1137-46.

S2. Lark DS, Torres MJ, Lin CT, Ryan TE, Anderson EJ, Neufer PD. Direct real-time quantification of mitochondrial oxidative phosphorylation efficiency in permeabilized skeletal muscle myofibers. Am J Physiol Cell Physiol. 2016;311:C239-45.

S3. Wittig I, Braun HP, Schagger H. Blue native PAGE. Nat Protoc. 2006;1:418-28.

S4. Jha P, Wang X, Auwerx J. Analysis of Mitochondrial Respiratory Chain Supercomplexes Using Blue Native Polyacrylamide Gel Electrophoresis (BN-PAGE). Curr Protoc Mouse Biol. 2016;6:1-14.

S5. Sabatini DD, Bensch K, Barrnett RJ. Cytochemistry and electron microscopy. The preservation of cellular ultrastructure and enzymatic activity by aldehyde fixation. J Cell Biol. 1963;17:19-58.

S6. Stringer C, Wang T, Michaelos M, Pachitariu M. Cellpose: a generalist algorithm for cellular segmentation. Nat Methods. 2021;18:100-6.

S7. Stringer C, Pachitariu M. Cellpose3: one-click image restoration for improved cellular segmentation. bioRxiv. 2024;

S8. Rueden CT, Schindelin J, Hiner MC, DeZonia BE, Walter AE, Arena ET, et al. ImageJ2: ImageJ for the next generation of scientific image data. BMC Bioinformatics. 2017;18:529.

S9. Shahtout JL, Eshima H, Ferrara PJ, Maschek JA, Cox JE, Drummond MJ, et al. Inhibition of the skeletal muscle Lands cycle ameliorates weakness induced by physical inactivity. J Cachexia Sarcopenia Muscle. 2024;15:319-30.

S10. Moorwood C, Liu M, Tian Z, Barton ER. Isometric and eccentric force generation assessment of skeletal muscles isolated from murine models of muscular dystrophies. J Vis Exp. 2013;e50036.

S11. Pande SV, Blanchaer MC. Carbohydrate and fat in energy metabolism of red and white muscle. Am J Physiol. 1971;220:549-53.

S12. Mogensen M, Sahlin K. Mitochondrial efficiency in rat skeletal muscle: influence of respiration rate, substrate and muscle type. Acta Physiol Scand. 2005;185:229-36.

S13. Perry CG, Kane DA, Lin CT, Kozy R, Cathey BL, Lark DS, et al. Inhibiting myosin-ATPase reveals a dynamic range of mitochondrial respiratory control in skeletal muscle. Biochem J. 2011;437:215-22.

S14. Picard M, Taivassalo T, Ritchie D, Wright KJ, Thomas MM, Romestaing C, et al. Mitochondrial structure and function are disrupted by standard isolation methods. PLoS One. 2011;6:e18317.
